# Nonoperative management of biliopleural fistula following living‐donor liver transplantation: A case report

**DOI:** 10.1002/ccr3.8210

**Published:** 2023-11-16

**Authors:** Kourosh Kazemi, Alireza Rasekhi, Sahar Sohrabi Nazari, Mohammad Mehdi Lashkarizadeh, Alireza Shamsaeefar, Mohammad Alikhani, Ali Akbari, Reza Shahriarirad

**Affiliations:** ^1^ Shiraz Transplant Center Abu Ali Sina Hospital, Shiraz University of Medical Sciences Shiraz Iran; ^2^ School of Medicine Shiraz University of Medical Sciences Shiraz Iran; ^3^ Thoracic and Vascular Surgery Research Center Shiraz University of Medical Science Shiraz Iran

**Keywords:** cholangiography, fistula, liver transplantation, pleural effusion

## Abstract

**Key Clinical Message:**

Biliopleural fistula is a rare but serious complication after liver transplantation that should be managed nonoperatively with antibiotics, pleural drainage, decompression of high‐pressure biliary tract, or ultimately surgery in unresponsive cases.

**Abstract:**

Bilious pleural effusion is a rare entity often iatrogenic, following hepatobiliary surgeries and biliary interventions, and has been reported only in a limited number of patients after liver transplantation. A 5‐year‐old girl underwent living donor liver transplantation due to progressive familial intrahepatic cholestasis. At the 7th day of the postoperative course, due to increased liver enzymes and bilirubin levels and intrahepatic bile duct dilatation on sonography, Magnetic Resonance Cholangiopancreaticography followed by a liver biopsy were performed; the findings demonstrated moderate intrahepatic bile duct dilatation and moderate cellular rejection associated with mild cholestasis, respectively. The patient was therefore administered a pulse of methylprednisolone; however, due to fever, peritonitis and also sonographic evidence of infected biloma collection adjacent to the transplanted liver, the patient underwent surgery. Laparotomy and peritoneal washout were performed and a Jackson‐Pratt drain was inserted adjacent to the liver cut surface. Succeeding tachypnea on 28th post day, led to detection of right side massive pleural effusion on chest Xray and hence thoracostomy tube was inserted. A diagnosis of biliopleural fistula was established and broad‐spectrum intravenous antibiotic therapy was started, followed by cholangiography, fistula closure, and bile duct stricture ballooning and internal‐external biliary catheter insertion. The patient was discharged in generally good condition on the 50th posttransplant day. The diagnosis of biliopleural fistula is facilitated with the utilization of chest imaging and pleural fluid analysis, however, a high index of suspicion is required.

## Graphical abstract

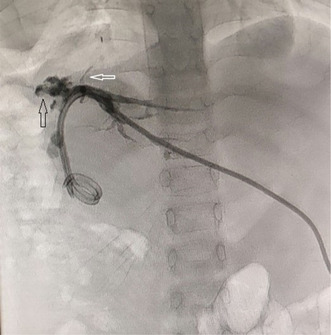

## 1 INTRODUCTION

Biliopleural fistula (BPF) is a rare condition that presents as pleural effusion and may develop congenital or following liver trauma, inflammatory and neoplastic processes, parasitic infections, or iatrogenic after hepatobiliary procedures.^1^ Bile contents can damage the lung and lead to ominous respiratory complications such as pneumonia, empyema, lung caustic injury, adhesion formation around the lung (entrapped lung), and even acute respiratory distress syndrome (ARDS). So, early detection is important in order to treat appropriately.

We describe a case of BPF that developed following living‐donor liver transplantation (LDLT) that was managed nonoperatively. Our purpose is to increase the awareness of surgeons and physicians dealing with transplant patients of this rare but serious complication.

## 2 CASE PRESENTATION

**FIGURE 1:**
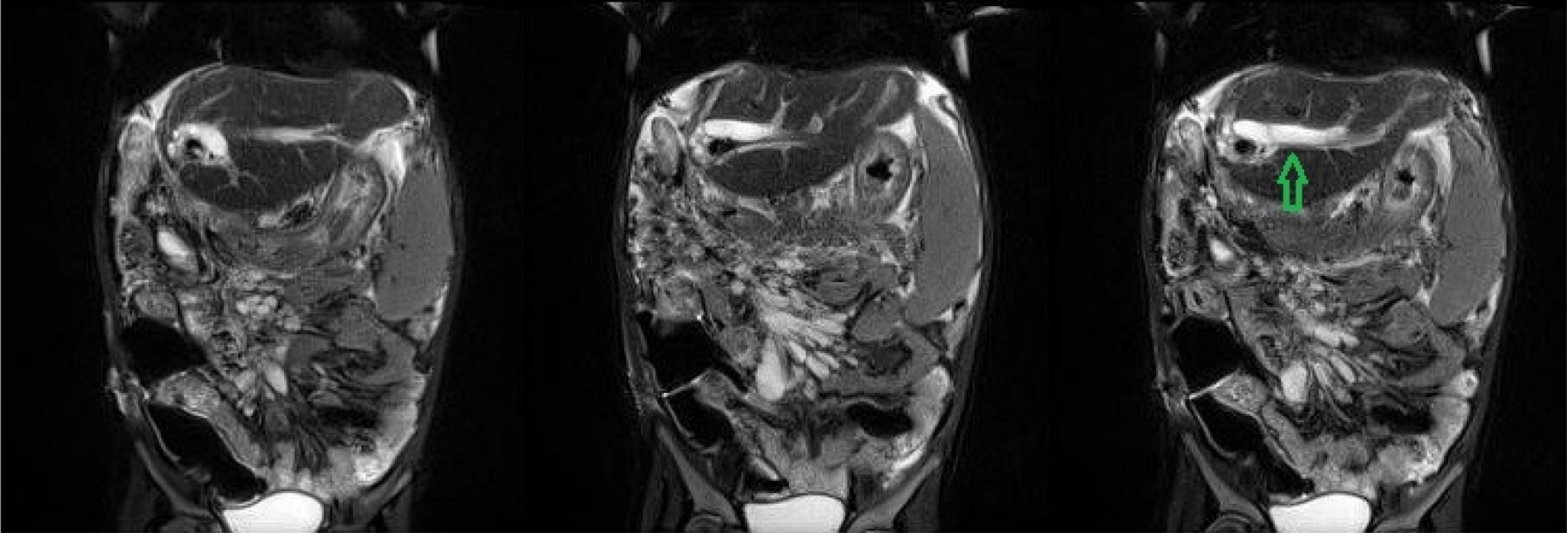
Magnetic Resonance Cholangiopancreatography (MRCP) shows moderate intrahepatic bile duct dilatation (green arrow).

A 5‐year‐old girl known history of progressive familial intrahepatic cholestasis (PFIC), presented with pruritus since 3 years, growth retardation (weight 13 Kg; under 5th percentile of weight for age) and recent abdominal distention. She also had a history of previous abdominal operation 1.5 year ago for exploring biliary system and liver biopsy that no biliary diversion was performed. She referred to our center for liver transplantation and underwent LDLT from her 26 years old mother. A left lateral segment of the donor's liver (single duct, single left hepatic artery and single portal vein) was transplanted and via Roux‐en‐Y hepaticojejunostomy anastomosis, the only draining duct was anastomosed to the Roux limb of jejunum. During the first postoperative week patient had an acceptable course but due to a cholestatic pattern of increased liver enzymes and bilirubin levels (Table 1), sonography of transplanted liver was done that revealed moderate intrahepatic bile duct dilatation with normal color doppler sonography, so Magnetic Resonance Cholangiopancreatography (MRCP) was performed on the 7th postoperation day, demonstrating moderate intrahepatic bile duct dilatation (Figure 1) but no clay stool was reported during her course.

**TABLE 1:** Course and value of the patient's liver function tests.

Laboratory test	Pretransplantation	Postoperation day			
		3rd	7th	28th	50th
Aspartate aminotransferase (IU/L)	156	55	96	28	28
Alanine transaminase (IU/L)	95	40	75	37	25
Alkaline phosphatase (IU/L)	1428	609	798	346	291
Total bilirubin (mg/dl)	18.17	10.35	10.49	0.83	0.59
Direct bilirubin (mg/dl)	9.55	3.39	5.57	0.37	0.26

Due to increased level of serum aminotransferases and bilirubin, liver biopsy was performed that showed moderate cellular rejection associated with mild cholestasis, so pulse of methylprednisolone was added to antibiotic therapy. During the second week after the transplantation, fever and abdominal pain developed and abdominal sonography revealed collection formation adjacent to the transplanted liver, along with the clinical impression of peritonitis, laparotomy was undertaken and a large volume of infected biloma (almost 300 mL) was detected adjacent to the liver cut surface and right subdiaphragmatic area without frank site of bile leakage. The peritoneal cavity was irrigated and a Jackson‐Pratt (JP) drain was inserted adjacent to the cut surface and it's daily drainage was subsequently measured and recorded.

**FIGURE 2:**
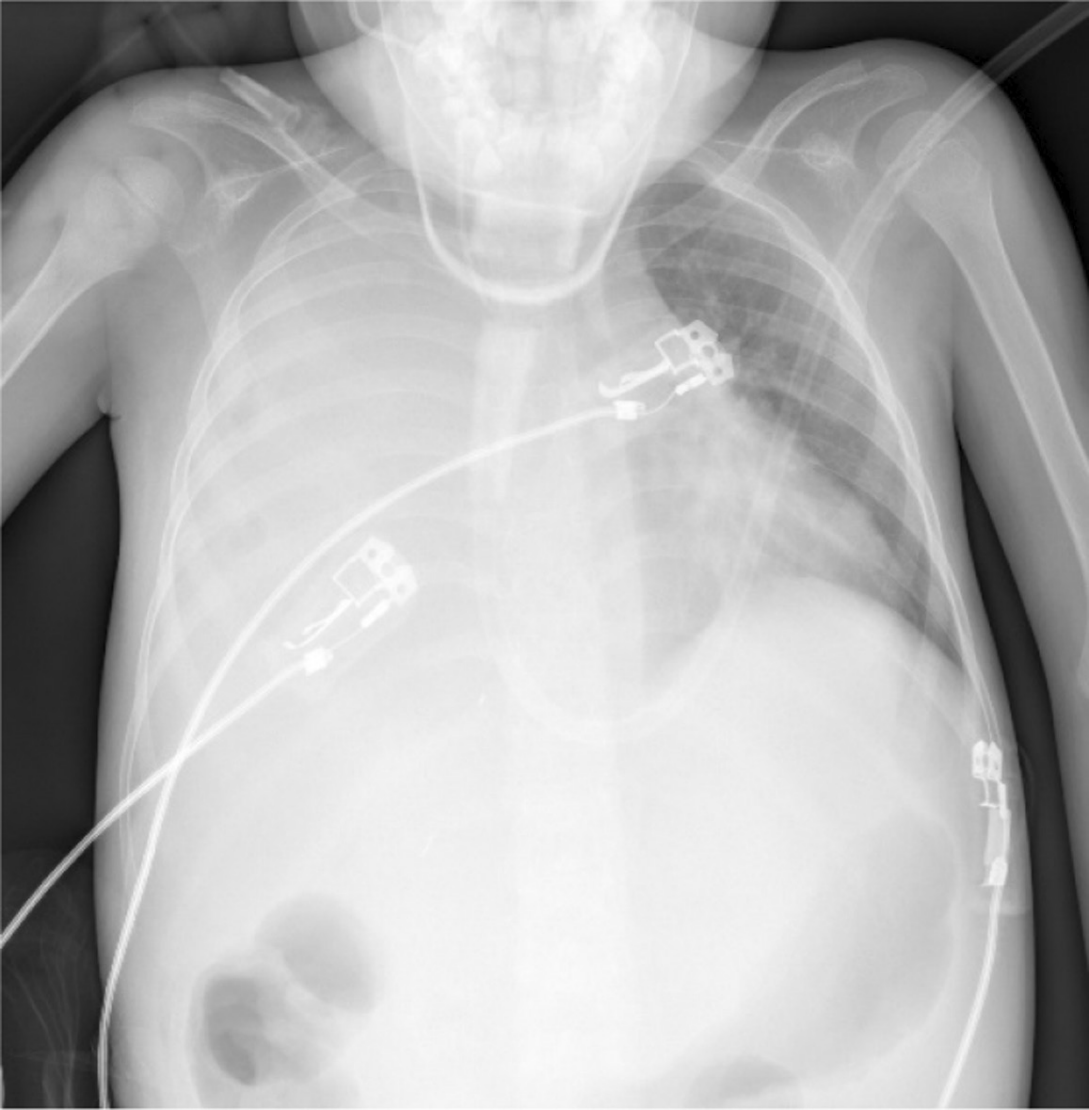
Chest roentgenogram demonstrating massive right side pleural effusion, due to biliopleural Fistula.

**FIGURE 3:**
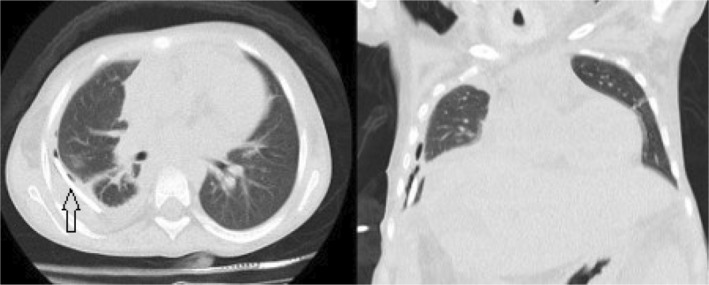
(Left Image) Axial computed tomographic image in soft tissue window demonstrating limited right side pleural effusion 3 days after thoracostomy drain insertion. (Right Image) Coronal computed tomographic image indicating pigtail drain in the right pleural cavity (black arrow).

Gradually drainage volume shifted toward zero but on the 28th postoperative day, she developed tachypnea without evidence of fever, cough, sputum, or chest pain. Chest X‐Ray (CXR) revealed a right‐side massive pleural effusion (Figure 2); therefore, a pleural pigtail was inserted under ultrasound (US) guide and 300 mL yellowish turbid (bilious) fluid was aspirated. Pleural fluid analysis revealed high bilirubin levels (pleural fluid total Bilirubin; 8.73, simultaneous serum total Bilirubin; 0.83) with neutrophil dominant leukocytosis (WBC count, 25,000; Neu, 98%) and fluid culture was positive for *Escherichia coli*. A suspicion of BPF was made and broad‐spectrum intravenous antibiotics (Imipenem & Linezolid) were continued. A chest computed tomography (CT) scan was done 3 days after the pigtail drain insertion, which showed less of a right‐side pleural effusion with accurate placement of the pigtail (Figure 3).

**FIGURE 4:**
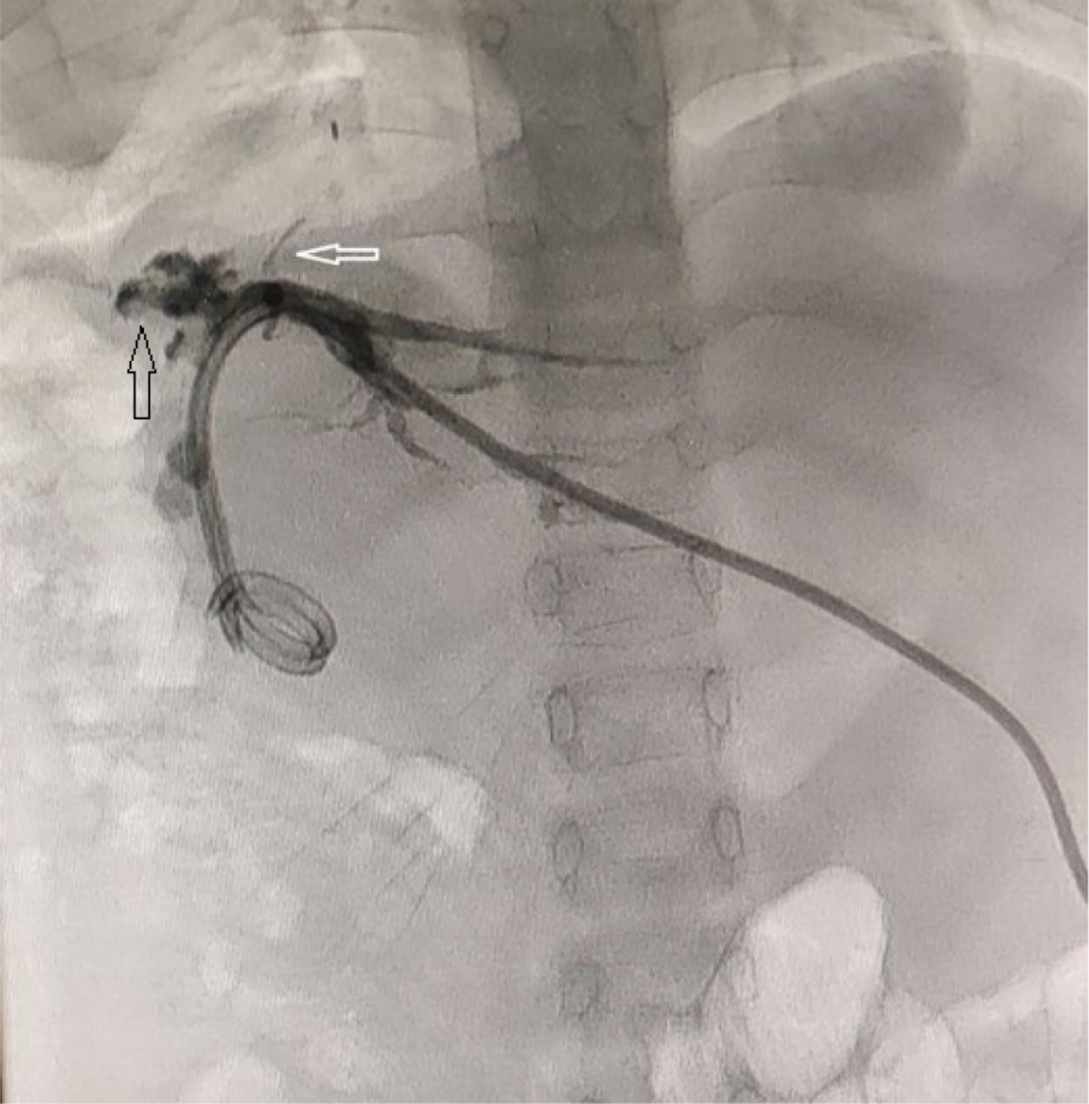
Image of the percutaneous transhepatic cholangiography demonstrating dilated intrahepatic bile ducts with stricture at the site of hepaticojejunostomy anastomosis and leakage from cut surface resulting in biliopleural fistula (white arrow). Bilio‐enteric continuity was seen with free passage of injected dye to the jejunal limb during cholangiography (black arrow).

Daily thoracostomy drain volume was 220 mL initially and declined to 40 mL after 2 weeks while the JP drain output was nil. It was hence deduced that there was some minor bile leakage from either the liver cut surface or the hepaticojejunostomy anastomosis. The mainstay of management of both scenarios is lowering the bile flow pressure, therefore the patient was candidate for cholangiography. Under US guide and conscious sedation after catheterization of dilated bile ducts of segments 2 and 3, Cholangiography was performed which revealed leakage from the allograft cut surface and some passage into the right hemithorax, plus bilioenteric continuity was confirmed with free passage of injected dye to the jejunal limb during cholangiography (Figure 4). Therefore, bile duct ballooning at the level of bilioenteric anastomosis was undertaken, and subsequently, the fistula ostium was closed with a mixture of glue and lipiodol. An internal‐external biliary draining catheter was also inserted. Following this procedure, the drainage output of the thoracostomy tube was decreased notably.

**FIGURE 5:**
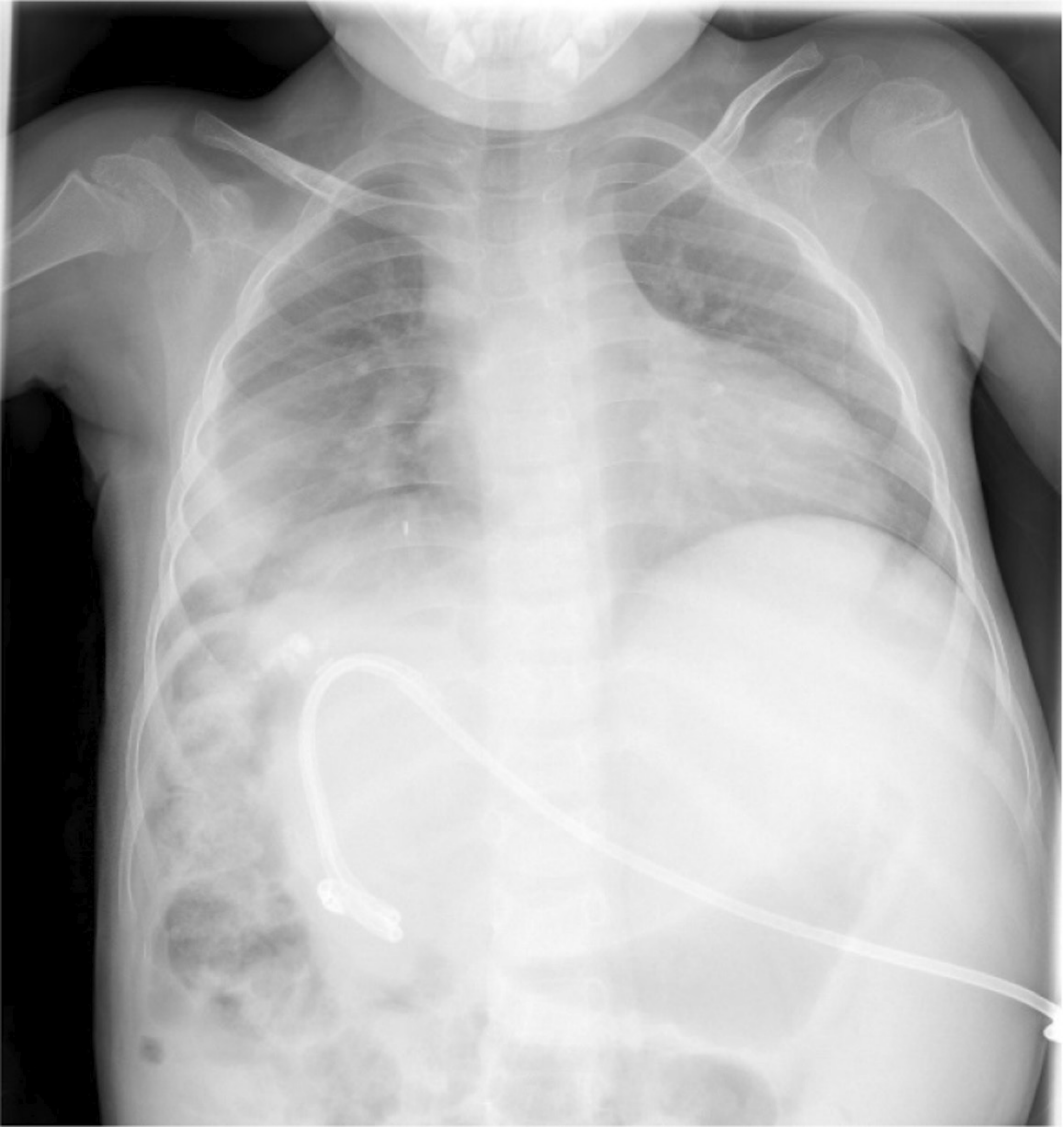
Chest X‐ray after thoracostomy tube removal.

The patient was discharged in generally good condition on the 50th posttransplant day with the thoracostomy tube in situ, and a decline in liver function tests (Table 1). On discharge, no intra‐abdominal collection was found in the US and the daily drain volume was less than 30 mL. She was followed with weekly visits as an outpatient in clinic. Gradually chest drainage fluid decreased and shifted toward zero in three subsequent weeks and finally the thoracostomy tube was removed following a satisfactory CXR. (Figure 5).

## 3 DISCUSSION

Bilious pleural effusion is a rare entity that tends to be mainly iatrogenic, following hepatobiliary surgeries and biliary interventions, and has been reported only in a limited number of patients following LTx. A brief review of reported cases of BPF and the formation of bilious pleural effusion following hepatobiliary interventions and surgeries are listed in Table 2.

**TABLE 2:** Review of reported cases of Biliopleaural fistula and the formation of bilious pleural effusion following hepatobiliary interventions and surgeries.

Hepatobiliary Procedure	Author(s)	Possible etiology	Treatment	Outcome
cholecystectomy	Aydogan et al.^2^	Diaphragmatic injury secondary to laparoscopic instruments	Thoracentesis, chest physiotherapy, analgesia & antibiotic therapy	Well response (discharge on the 7th POD)
	Prabhu et al.^3^	N/A	Intercostal pleural drain, PTBD, bilio‐enteric bypass	N/A
Gallbladder injury during laparotomy	Jenkinson et al.^4^	Perforation in the Hartmann's pouch of the gallbladder during laparotomy (1 week ago)	Thoracentesis, laparotomy, peritoneal washout & cholecystectomy	Well response
Hepaticojejunostomy anastomosis	Basu et al.^5^	Bile leakage & peritonitis	Intercostal pleural drain, ventilatory support	Pleural drainage decreased but patient's condition deteriorated (ARDS & MODs) and she died on the 21th POD
Blunt abdominal Trauma	Cooper et al.^6^	Liver laceration grade V	Thoracostomy tube insertion	Well response during 48 hours (F/U till post‐trauma day 36)
Penetrating abdominal trauma	Dahiya et al.^7^	Gunshot injury	Chest tube insertion, ERCP, sphincterotomy, biliary stenting, pigtail drainage of subphrenic collection, antibiotics	Well response (30 months F/U)
	Tesfaye et al.^8^	Gunshot injury (bullet tract)	Thoracotomy, repair of liver laceration & diaphragmatic defect, lung decortication	Well response
	Ball et al.^9^	Infected biliary abscess eroding the diaphragmatic repair (recent laparotomy due to abdominal gunshot)	Right thoracoabdominal incision, washout, excision of fistula tract & wedge resection of Rt lung lower lobe, chest tube & peritoneal drains insertion, ERCP, biliary stenting	Well response (the drainage stopped on the 8th POD)
PTC & biliary Drainage	Singh et al.^1^ 8 cases	Amoebic liver abscess (3 cases), pyogenic liver abscess (1 case), thoracoabdominal trauma (3 cases), iatrogenic (PTC) (1 case)	Low fat diet, Octreotide, antibiotics, tube thoracostomy, percutaneous drainage of abdominal abscess, ERCP, sphincterotomy (5 successful cases) or surgical closure of fistula (2 unsuccessful cases for endoscopic sphincterotomy) or choledochojejunostomy (1 case)	Complete recovery (mean F/U; 26 months)
	Strange et al.^10^ 3 cases	Complete biliary obstruction, catheter placement between the 9th & 10th ribs in the midaxillary line, prolonged drainage	⧠ Early reinstitution of biliary drainage, pleural drainage, antibiotic therapy (2 cases) ⧠ Thoracentesis, ERCP, internal biliary drainage & PTBD catheter removal (1 case)	Complete recovery
	Gao et al.^11^	Percutaneous transhepatic cholangiodrainage & irradiation stent insertion	Pleural drainage, ERCP, fistula closure with transcatheter coil embolization	Well response (2 months F/U)
	Lee et al.^12^	The catheter passed through the pleural cavity before crossing the diaphragm	Pleural drainage & antibiotic therapy	Relieved over 2 weeks
	Al‐Qahtani^13^	Pleural cavity can be traversed during the procedure to gain access to the biliary tract, Elevated pressure gradient in the biliary tract (obstructive jaundice), long duration of catheter in place	Chest tube insertion, video assisted thoracoscopic evacuation of the pleural effusion, lung decortication	Well response (4 months F/U)
	Turkington et al.^14^	The catheter became dislodged with the tip in hepatic bile duct while the side‐holes in pleural cavity	Intercostal pleural drain & percutaneous bilious stenting (inserted across the biliary stricture)	N/A
	Yi‐Yung Yu et al.^15^	Traversing the pleura by the catheter before entering the bile duct	Tube thoracostomy, antibiotics, PTBD tube, surgical repair of biliopleural fistula (due to unsuccessful conservative management)	The patient expired 5 days after surgery (respiratory failure)
	Bilal et al.^16^	Following PTBD insertion	Chest tube insertion, antibiotics, continued use of PTBD	N/A
	Kim & Zangan^17^	Fistula formation when the side holes of the catheter are outside the bile duct in the pleural cavity	Thoracentesis, tunneled pleurX catheter placement, cholangiography and upsized PTBD	Despite the satisfactory biliary diversion & pleural drainage, the patient's clinical course worsened and he died in the hospital
	De Meester et al.^18^	Probably the path created by the large drainage tubes which bile could leak back into the pleural cavity in the presence of persistent biliary tract obstruction	Tube thoracostomy, ERCP & removal of bile duct stones, nasobiliary catheter inserted into the Rt hepatic duct	Complete recovery
ERCP	Van Niekerk et al.^19^	Biliary tract perforation	The patient refused treatment	Due to underlying metastatic gallbladder cancer, the patient elected to receive hospice care
Percutaneous RFA of liver metastasis	Pende et al.^20^	Diaphragmatic thermal injury & intrahepatic biloma	Tube thoracostomy, ERCP & sphincterotomy, nasobiliary drainage positioned above the fistula, percutaneous drain insertion in liver purulent biloma, ERCP redo & plastic biliary endoprosthesis placement & NBD removal	Well response & recovery
TACE of malignant liver lesions	Delis et al.^21^	Subphrenic liver abscess formation and rupture of it in the pleural space	Chest tube insertion, ERCP & silastic stent placement, intravenous hyperalimentation	Despite successful management of pleural drainage, the patient succumbs from sepsis & liver failure 15 days later
Liver metastatectomy	Hamers et al.^22^	Diaphragmatic injury during operation	Antibiotics, tube thoracostomy, percutaneous subdiaphragmatic drain, ERCP & stenting	The patient discharged on day 49 in a faily good condition with an abdominal drain in situ just producing some yellowish fluid
	Kerawala & Jamal^23^	Bile leakage and collection	Chest tube insertion, antibiotics, ERCP & sphincterotomy	Resolved over 23 days
Liver transplant	Karnik & Shair^24^	Bile leakage at the anastomotic site of the liver transplant	Chest tube insertion, antibiotics, ERCP & biliary stent placement	Resolved 2 weeks after intervention
	López‐Garnica et al.^25^	High intraductal pressure due to biliary stenosis	Pleural drainage, ERCP, sphincterotomy & plastic stent placement then ERCP redo 2 months later, stent removal, ballooning & stent replacement	Resolved over 2 months

Abbreviations: ARDS, acute respiratory distress syndrome; ERCP, endoscopic retrograde cholangiopancreatography; F/U, follow‐up; MODs, multi‐organ dysfunctions; N/A, Not available; NBD, nasobiliary drainage; POD, postoperative day; PTBD, percutaneous transhepatic biliary drainage; PTC, percutaneous transhepatic cholangiography; RFA, radiofrequency ablation; TACE, transarterial chemoembolization.

Previously, Karnik et al. reported bilothorax following orthotopic LTx in a 62‐year‐old male with hepatocellular carcinoma and hepatitis B virus, who presented with a massive right‐sided pleural effusion, in which drainage of the pleural fluid along with endoscopic retrograde cholangiopancreatography (ERCP) revealed bile leak at the biliary anastomotic site. The case was managed with biliary stent placement.^26^ López‐Garnica et al.^25^ also reported a 44‐year‐old male patient with nonalcoholic fatty liver disease and cirrhosis due to methotrexate use, who underwent LTx from a deceased donor, presenting with abdominal pain, nausea, and diarrhea 1 month after surgery. The patient was diagnosed with a case of BPF based on pleural effusion drainage consistent with bile fluid characteristics. The patient was managed via ERCP with sphincterotomy and internal plastic stent placement into the biliary tree. However, the patient presented with jaundice 2 months after stent placement due to stenosis and was eventually treated with balloon dilatation and biliary stent replacement.^25^

Various theories have been explained as the cause of bilious pleural effusion following LTx, the most probable of which are:

- Biliary outflow stricture and obstruction following LTx with high‐pressure gradient in the biliary tract results in;
- Flowing bile into low‐pressure spaces, such as the thoracic cavity with negative pressure, and finally bilious pleural effusion forms^27^ and
- Subdiaphragmatic collection formation led to inflammation & erosion through the diaphragm and BPF forms.^28^
- Intraperitoneal fluid has been transmitted via intradiaphragmatic pleuroperitoneal pores and finally bilious pleural effusion forms.
- Undiagnosed intraoperative diaphragmatic injury, which is less probable because of evidence of pneumothorax, results in BPF.

Our case had a history of previous abdominal surgery with a lot of adhesions being encountered during the transplant procedure mostly in the right upper quadrant of the abdomen. As adhesiolysis was done mainly using electrocautery and the argon beam coagulator, this may have resulted in ischemia and micro‐damage in less vascularized tendinous part of diaphragm which along with the postoperative intra‐abdominal infection may have increased the risk of fistula formation.

Taking into account all of these considerations, no consensus was reached regarding the precise cause of BPF in our case. Overall, although the diagnosis of BPF is facilitated with the utilization of chest imaging and pleural fluid analysis, a high index of suspicion is required. We were able to manage the case based on a nonoperative approach via broad‐spectrum intravenous antibiotic therapy, followed by cholangiography, fistula closure, and bile duct stricture ballooning.

The mainstay of treatment of BPF is a conservative approach including evacuation of bilious fluid from the pleural cavity (tube thoracostomy), decompression of high‐pressure biliary tract (ballooning and stent placement and endoscopic sphincterotomy), percutaneous drainage of intraperitoneal collection, and antibiotic administration while the closure of the fistula (at first nonsurgically, then surgically) remains for patients unresponsive to nonoperative approach.^26^, ^28^

## 4 CONCLUSION

We report a pediatric patient with BPF following LDLT who was treated with a multidisciplinary nonoperative approach. BPF is a rare complication of liver transplantation can be associated with considerable respiratory complications. The diagnosis of BPF is facilitated with the utilization of chest imaging and pleural fluid analysis, however a high index of suspicion is required. Use of electrocautery and argon beam coagulator for hemostasis may result in ischemia of hemidiaphragm, and should be considered as an additional hypothetical factor for BPF formation.

## AUTHOR CONTRIBUTIONS

**Kourosh Kazemi:** Conceptualization. **Alireza Rasekhi:** Conceptualization; investigation. **Sahar Sohrabi Nazari:** Data curation. **Mohammad Mehdi Lashkarizadeh:** Data curation. **Alireza Shamsaeefar:** Data curation. **Mohammad Alikhani:** Data curation. **Ali Akbari:** Validation. **Reza Shahriarirad:** Conceptualization; writing – original draft; writing – review and editing.

## FUNDING INFORMATION

No financial support was received for this case report.

## CONFLICT OF INTEREST STATEMENT

The authors declare that they have no competing interests.

## ETHICS STATEMENT

Written informed consent was obtained from the patient's parents in our study. The purpose of this research was completely explained to them and they were assured that their information will be kept confidential by the researcher. The present study was approved by the Medical Ethics Committee of the academy.

## CONSENT

Written informed consent was obtained from the patient's parents for publication of this case report and any accompanying images. A copy of the written consent is available for review by the Editor of this journal.

## Data Availability

All data regarding this study has been reported in the manuscript. Please contact the corresponding author if you are interested in any further information.
